# Comparative Transcriptomics Identifies Neuronal and Metabolic Adaptations to Hypergravity and Microgravity in *Caenorhabditis elegans*

**DOI:** 10.1016/j.isci.2020.101734

**Published:** 2020-11-25

**Authors:** Craig R.G. Willis, Nathaniel J. Szewczyk, Sylvain V. Costes, Ingrid A. Udranszky, Sigrid S. Reinsch, Timothy Etheridge, Catharine A. Conley

**Affiliations:** 1 Department of Sport and Health Sciences, College of Life and Environmental Sciences, University of Exeter, Exeter, EX1 2LU, UK; 2 MRC-ARUK Centre for Musculoskeletal Ageing Research and National Institute of Health Research, Biomedical Research Centre, School of Medicine, Royal Derby Hospital, University of Nottingham, Derby, DE22 3DT, UK; 3 Ohio Musculoskeletal and Neurological Institute (OMNI) and Department of Biomedical Sciences, Ohio University, Athens, OH 43147, USA; 4 Space Biosciences Division, NASA Ames Research Center, Moffett Field, CA 94035, USA; 5 Lockheed Martin Space Operations, Moffett Field, CA 94035, USA; 6 Space Science and Astrobiology Division, NASA Ames Research Center, Moffett Field, CA 94035, USA

**Keywords:** Neuroscience, Transcriptomics, Space Sciences

## Abstract

Deep space exploration is firmly within reach, but health decline during extended spaceflight remains a key challenge. In this study, we performed comparative transcriptomic analysis of *Caenorhabditis elegans* responses to varying degrees of hypergravity and to two spaceflight experiments (ICE-FIRST and CERISE). We found that progressive hypergravitational load concomitantly increases the extent of differential gene regulation and that subtle changes in ∼1,000 genes are reproducibly observed during spaceflight-induced microgravity. Consequently, we deduce those genes that are concordantly regulated by altered gravity *per se* or that display inverted expression profiles during hypergravity versus microgravity. Through doing so, we identify several candidate targets with terrestrial roles in neuronal function and/or cellular metabolism, which are linked to regulation by *daf-16*/FOXO signaling. These data offer a strong foundation from which to expedite mechanistic understanding of spaceflight-induced maladaptation in higher organisms and, ultimately, promote future targeted therapeutic development.

## Highlights

• Comparative transcriptomics in *C. elegans* exposed to hypergravity and spaceflight
• Bioinformatics identifies novel putative regulators of altered gravitational load
• Candidate molecules infer a close gravity > *daf-16*/FOXO > neuronal link

## Graphical Abstract

**Figure:**
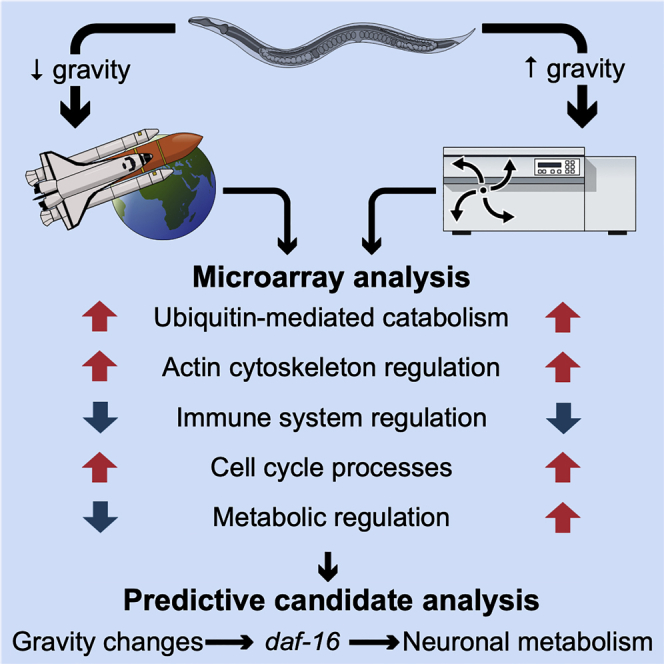

## Introduction

Living systems on earth have evolved to function optimally at unit gravity (1 × *g*). Exposure to altered gravity, as with hypergravity (>1 × *g*) or microgravity (∼0 × *g*), can subsequently lead to a multitude of physiological changes, with the musculoskeletal, cardiovascular, endocrine, immune, and nervous systems all adversely impacted (Demontis et al., 2017; Frett et al., 2016; Genchi et al., 2016). The consequent risk that altered gravity environments pose to whole-body health is most relevant to space exploration: astronauts are exposed to “hypergravitational” forces due to acceleration during take-off and landing (Frett et al., 2016), as well as microgravity in-flight (Thirsk et al., 2009). Furthermore, it is probable that other colonizable planets beyond Earth will have inertial conditions that deviate from unit gravity (Kalb and Solomon, 2007; Kaltenegger et al., 2011). Overcoming the health challenges associated with altered gravity would thus help accelerate safe human space travel and habitation, both of which remain key aims of the world's space agencies (Crusan et al., 2017; Institute of Medicine, 2008).

A crucial step toward overcoming any pathophysiological condition is first defining its underlying molecular mechanism(s). However, understanding of the molecular gravity phenotype directly in humans remains limited by the clear technical, operational, and economic challenges of large-scale micro- and hypergravity human studies (e.g., high cost, low numbers of available participants, feasibility of tissue sampling). Rodents flown in space are a good alternative to humans and have helped to aid understanding on some of the transcriptome-wide changes that occur as a result of spaceflight (e.g., Beheshti et al., 2018a; Blaber et al., 2017; Gambara et al., 2017), but experiments have generally been limited in size to 20 or fewer flight animals onboard the International Space Station (ISS) or the Space Shuttle (Beheshti et al., 2018b, Beheshti et al., 2019; Ray et al., 2019), owing to space and crew time limitations. Use of smaller yet biologically relevant model organisms circumvents many of these obstacles. For example, the nematode *Caenorhabditis elegans* (*C. elegans*) represents a primary model for space life sciences owing to its small size, short life span, ease of culture, low expense (Brenner, 1974), and a completely defined genome (C. elegans Sequencing Consortium, 1998) with strong evolutionary conservation in humans (Lai et al., 2000). Previous work demonstrates *C. elegans* are capable of successful reproductive cycles in both microgravity (Oczypok et al., 2012) and hypergravity (Qiao et al., 2013; Saldanha et al., 2016) and survive even when exposed to hypergravitational forces upward of 400,000 × *g* (de Souza and Pereira, 2018). Moreover, in microgravity *C. elegans* display molecular signatures (e.g., impaired insulin and cell adhesion signaling) and physiological features (e.g., reduced movement capacity) that closely mirror those observed in humans (Higashibata et al., 2006, 2016; Nichols et al., 2006; Selch et al., 2008). As such, defining precise molecular adaptations to altered gravity in *C. elegans* should ultimately provide the foundations for progressing understanding on the mechanisms of spaceflight-induced health decline in mammals and, eventually, humans.

Micro- and hypergravity exposures represent opposing ends of the gravity spectrum and each associate with physiological adaptations that can be mirror opposites (e.g., divergent changes in collagen biosynthesis; Seitzer et al., 1995) and/or demonstrate a continuum response across micro- and hypergravity environments (e.g., tissue metabolism; Plaut et al., 2003). It is, therefore, logical to postulate that the most robust gravity-responsive transcriptional profiles of microgravity might be the inverse of the hypergravity expression pattern. Conversely, certain gravity phenotypes overlap (e.g., suppressed immunity and thyroid cell decline; Albi et al., 2012). Thus, unidirectional gene signatures common to micro- and hypergravity might provide insight into the most prominent maladaptations to altered gravity *per se*. Transcriptomic databases provide one means to contrast the microgravity versus hypergravity response. For example, the “International *C. elegans* Experiment FIRST” (ICE-FIRST) and “*C. elegans* RNA Interference in Space Experiment” (CERISE) experiments (Higashitani et al., 2009; Szewczyk et al., 2008) performed microarray analysis on worms following spaceflight microgravity exposure onboard the International Space Station (Adenle et al., 2009; Higashibata et al., 2016). Although a robust and complete transcriptomic signature of flight adaptation in *C. elegans* remains elusive, comparison within/between experiments illustrates operational- and dietary-independent, but generational-dependent, spaceflight-induced changes in genes involved in muscle contraction and energy metabolism (Etheridge et al., 2015; Higashibata et al., 2016). Additionally, although transcriptional responses to hypergravity have been studied in human Jurkat T cells (Thiel et al., 2020), fruit flies (Hateley et al., 2016) and a small number of rodent tissues (Ishizawa et al., 2009; Kawao et al., 2020; Lee et al., 2015; Pulga et al., 2016), consensuses on the hypergravity versus spaceflight microgravity response are lacking (Casey et al., 2015; Kopp et al., 2018), the complete delineation of which would serve a useful basis for developing safe hypergravity-based loading interventions to counter health decline during space travel.

This study therefore aimed to determine the reproducible transcriptional profiles of altered gravity in *C. elegans* using data hosted in NASA's GeneLab Repository. Additionally, we exploit the power of predictive network and transcription factor analyses as biologically driven tools for deriving candidate molecular drivers of gravity responsiveness. The resultant transcriptional signatures should promote hypothesis generation for future mechanistic understanding of, and countermeasures against, the maladaptive health consequences of extended spaceflight.

## Results

### Dataset Overview

A total of four independent microarray datasets were included herein, as outlined in Table 1. One such dataset (GLDS-190, Szewczyk et al., n.d.) contained expression data from *C. elegans* exposed to either normal (1 × *g* control) or hypergravitational (5 × *g*, 10 × *g* or 15 × *g*) forces for 4 days via centrifugation. The remaining three datasets (GLDS-113, Higashibata and Hashizume, n.d.a; GLDS-112, Higashibata and Hashizume, n.d.b; GLDS-41, Higashibata and Hashizume, n.d.c) each included transcriptomic profiling of *C. elegans* exposed to ground gravitational levels (1 × *g* ground control) on earth or spaceflight-induced microgravity on the ISS. In particular, GLDS-113 contains transcriptomic data from the ICE-FIRST experiment, whereas GLDS-112 and GLDS-41 each contain expression data from the more recent CERISE experiment (4 and 8 days, respectively). After appropriate data pre-processing (see Transparent Methods), a total of 9,761 genes were found to be present in all four datasets and therefore were used as the basis for downstream analyses.

**Table 1:** Overview of all Hypergravity and Spaceflight Microgravity Studies from which Microarray Data Have Been Included in the Current Work

Study Code	Condition	Mission	Duration (days)	Strain	Life Stage	Culturing
GLDS-190	Hypergravity	NA	4	CC1	Mixed	CeMM
GLDS-113	Microgravity	ICE-FIRST	10	N2	Mixed	CeMM
GLDS-112	Microgravity	CERISE	4	N2	Adult	Liquid
GLDS-41	Microgravity	CERISE	8	N2	Adult	Liquid

### Transcriptomic Changes in *C. elegans* Exposed to Increasing Hypergravity

**Figure 1:**
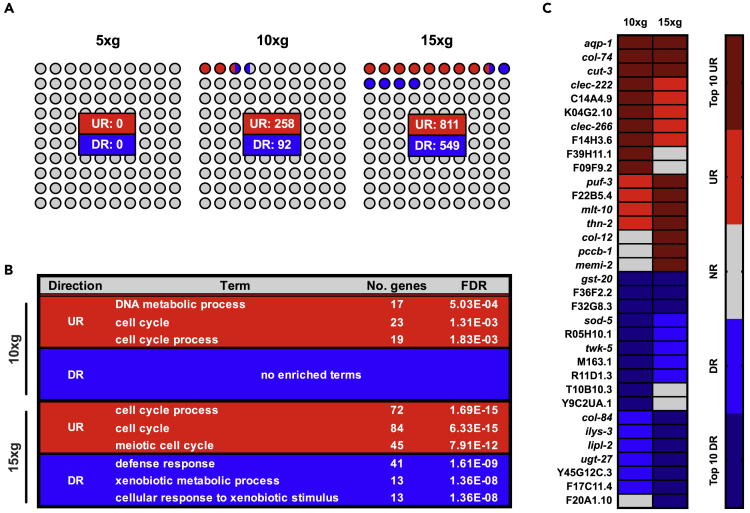
Transcriptomic Response of *C. elegans* to Increasing Hypergravitational Load (A) Number of upregulated (UR) and downregulated (DR) genes for each level of hypergravity versus 1 × *g* controls (FDR ≤10%). Dots depict proportion of genes in each case that are upregulated (red), downregulated (blue), or not regulated (gray) by hypergravity. (B) Top three enriched Gene Ontology Biological Process terms for genes differentially regulated by 10 × *g* or 15 × *g*. (C) Top 10 ranked genes (by FDR) in each of the 10 × *g* and 15 × *g* differential gene lists (UR and DR). See also Tables S1, S2, and S3.

We first considered the transcriptional alterations that occur in *C. elegans* during hypergravity, with particular emphasis on the effect of progressively increasing hypergravitational load. The GLDS-190 dataset was utilized to infer differential expression in worms subjected to different hypergravitational forces versus corresponding 1 × *g* controls. Increasing hypergravitational load resulted in a concomitant increase in the number of differentially expressed genes: zero genes were differentially expressed above the cut-off threshold used in this study during 5 × *g* forces, whereas 350 genes showed differential expression in animals exposed to 10 × *g* and 1,360 genes were differentially expressed during 15 × *g* hypergravity (Figure 1A). “Cell cycle” and “cell cycle process” were among the most significantly enriched Gene Ontology (GO) Biological Process (BP) terms for upregulated genes in 10 × *g* and 15 × *g* conditions (Figure 1B), with their top-ranked up-/downregulated gene lists also sharing several of the same genes (Figure 1C). A list of genes that were expressed with statistically significant differences over all analyses herein is provided in Table S1, whereas all associated enriched GO BP terms are given in Table S2.

**Figure 2:**
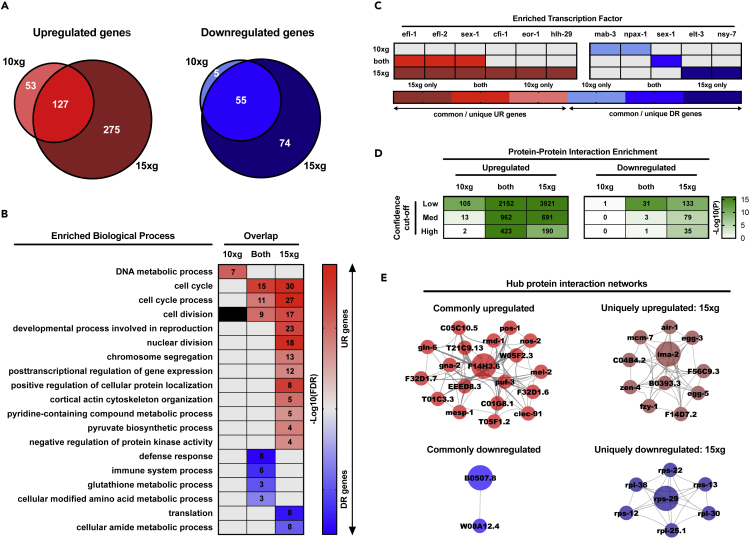
Comparison of the 10 × *g* versus 15 × *g* Hypergravity Transcriptomes (A) Venn diagrams depicting the degree of overlap between genes differentially up- or downregulated by 10 × *g* versus 15 × *g* hypergravity exposure, as based on the rank-rank hypergeometric overlap (RRHO) analysis. (B) Non-redundant, enriched Gene Ontology Biological Process terms for each possible overlap. Number of genes enriched in a given term are provided within associated boxes of the heatmap. Darker shading denotes greater significance. (C) Predicted transcription factors of each common-/uniquely regulated gene set. (D) Quantity (in boxes) and significance (green shading scale) of enriched protein-protein interactions among the genes of each possible overlap, across a range of interaction “confidence” cutoffs. (E) Hub proteins (and their interactions) for commonly up-/downregulated genes, as well as genes uniquely regulated by 15 × *g* gravity (relative to 10 × *g*). Largest node depicts top-ranked hub protein. See also Tables S1, S2, and S3.

To pursue the above-mentioned observations further, we overlaid 10 × *g* and 15 × *g* differentially expressed genes to compare the transcriptional profiles of each hypergravitational load in direct relation to one another (Figure 2A). Doing so demonstrated that (1) the majority of genes differentially regulated during 10 × *g* are similarly regulated by 15 × *g*, and (2) 15 × *g* is further characterized by a distinct set of expression changes beyond 10 × *g*. Genes upregulated by both 10 × *g* and 15 × *g* were enriched exclusively for cell cycle-related GO terms, with genes uniquely upregulated by 15 × *g* involved in processes such as “nuclear division” and “pyruvate biosynthetic process” (Figure 2B). Commonly downregulated genes included those involved in the innate immune response, whereas genes involved in translation were uniquely downregulated by 15 × *g* (Figure 2B). Protein networks constructed from common and 15 × *g*-specific hypergravity gene lists were also enriched with strong interactions (confidence >0.4; Figure 2D), providing additional evidence for coherent biological functioning.

These networks were next examined for their central (highly connected) “hub” components (Figure 2E), to establish mechanistic targets of common (10 × *g* and 15 × *g*) and 15 × *g*-specific hypergravitational regulation. Notably, hub components of 15 × *g*-specific downregulation were exclusively ribosomal protein subunits, whereas the top hub component of 15 × *g*-specific upregulation was identified as *ima-2*, which serves to facilitate nuclear localization sequence-bearing protein import into the nucleus (UniProt Consortium, 2019). Hub statistics for all analyses herein are given in Table S3. We further expanded candidate target identification by testing the enrichment of common/uniquely regulated gene sets for putative transcription factors (TFs) (Figure 2C). Among those most prevalent were *efl-1* and *efl-2*; both E2F-like TFs predicted to regulate genes either commonly upregulated by 10 × *g*/15 × *g* or by 15 × *g* alone.

### Reproducible Transcriptomic Changes in Space-Flown *C. elegans*

**Figure 3:**
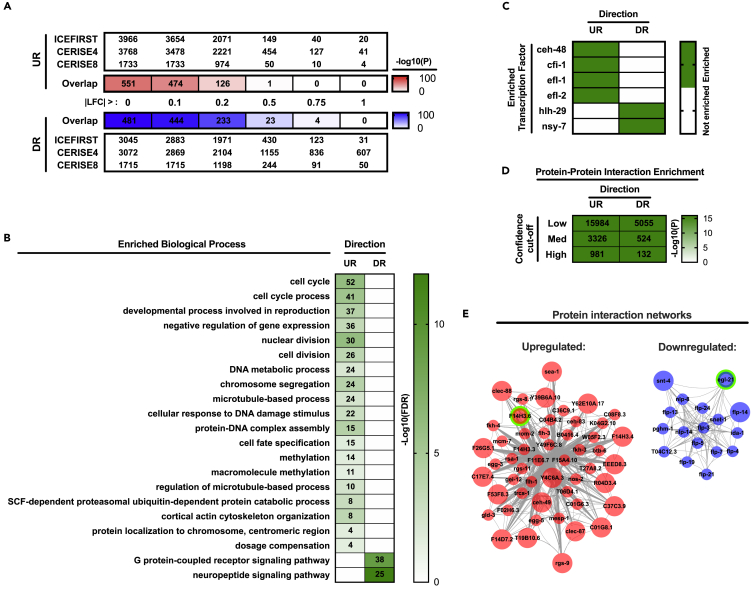
Reproducible Gene Expression Changes during Spaceflight (A) Overlap of differentially expressed genes (FDR ≤10%) across each of the three spaceflight studies. Red/blue shading denotes significance of the corresponding overlap for upregulated (UR) and downregulated (DR) genes, respectively. (B) Non-redundant, enriched Gene Ontology Biological Process terms for common UR/DR genes across microgravity studies (defined by FDR ≤10% and |LFC| > 0). Number of genes enriched in a given term are provided within associated boxes of the heatmap. Darker shading denotes greater significance. (C) Predicted transcription factors of UR and DR gene sets. (D) Quantity (in boxes) and significance (green shading scale) of enriched protein-protein interactions among UR and DR gene lists, across a range of interaction “confidence” cutoffs. (E) Protein interactions (confidence >0.15) for UR and DR genes with a hub score >0.6. Larger nodes depict “hub” proteins (hub score >0.8), with top-ranked hub protein in each case identified by a green node border. See also Tables S1, S2, and S3.

We next sought to establish consistent transcriptional signatures in *C. elegans* exposed to spaceflight-related microgravity, independent of potentially confounding external and/or experimental factors. We therefore integrated expression data from the ICE-FIRST and CERISE spaceflight experiments, across which exist distinct operational, dietary, and generational differences (Table 1). Very little overlap was observed between large-scale log2 fold-changes (LFC) (|LFC| > 0.5) in expression (versus 1 × *g* ground control) specific to each spaceflight dataset (Figure 3A). However, strong overlap was found when also taking into account smaller-scale expression changes (i.e., |LFC| > 0) (Figure 3A), suggesting that reproducible transcriptomic changes in space-flown worms are subtle, as previously reported (Higashibata et al., 2016; Selch et al., 2008). In the instance of differentially regulated genes with |LFC| > 0, genes uniformly downregulated during spaceflight were predominantly involved in neuropeptide- and/or G protein-related signaling, whereas those consistently upregulated were enriched for processes related to cell cycle and DNA modification, as well as microtubule regulation and ubiquitin-dependent protein catabolism (Figure 3B).

Protein networks for genes coherently upregulated/downregulated during microgravity were found to be highly enriched with protein-protein interactions, even when only considering very strong interactions (confidence >0.7; Figure 3D). The top hub within the upregulated microgravity network was identified as the as yet uncharacterized protein F14H3.6, whereas the top hub of the downregulated microgravity network was *egl-21* (a major carboxypeptidase) (Figure 3E). Consistent with the upregulated hypergravity signature, genes upregulated during microgravity were also highly enriched for being under the predicted control of *efl-1* and *efl-2* TFs (Figure 3C). Moreover, the most enriched TF of downregulated microgravity genes was *nsy-7*, which serves to function in determining left/right neuronal asymmetry (UniProt Consortium, 2019).

### Comparison of the Worm Transcriptome during Hypergravity Versus Microgravity

Finally, we explored the degree to which differentially expressed genes during microgravity and hypergravity overlap. Specifically, we compared reproducible microgravity gene changes with 15 × *g* hypergravity gene changes, since (1) 15 × *g* induces the greatest number of transcriptomic perturbations, and (2) the 15 × *g* transcriptional profile encapsulates the majority of 10 × *g* transcriptional changes.

**Figure 4:**
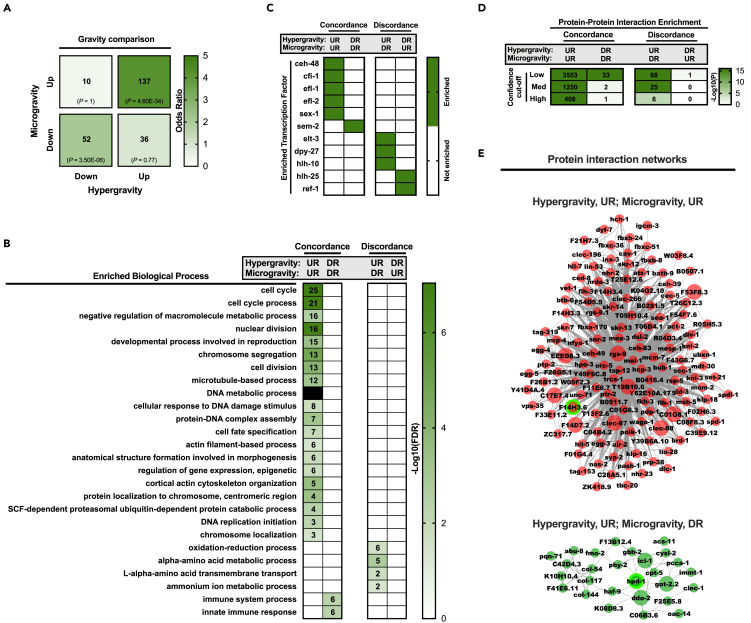
Concordant and Discordant Gene Changes with Micro- and Hypergravity (A) Overlap of differentially expressed hypergravity genes (15 × *g*) and reproducibly differentially expressed microgravity genes (|LFC| > 0). Green shading denotes strength of Fisher's exact odds ratio, with corresponding significance and number of overlapping genes provided within each corresponding box. (B) Non-redundant, enriched Gene Ontology Biological Process terms for each possible hypergravity versus microgravity overlap permutation. Number of genes enriched in a given term are provided within associated boxes of the heatmap. Darker shading denotes greater significance. (C): Predicted transcription factors of each overlapping gene set. (D): Quantity (in boxes) and significance (green shading scale) of enriched protein interactions among genes of each possible overlap, across range of interaction “confidence” cutoffs. (E) Protein interactions (confidence >0.15) for genes commonly upregulated with hypergravity/microgravity, as well as genes upregulated with hypergravity but downregulated by microgravity. Larger nodes depict “hub” proteins (hub score >0.8), with top-ranked hub protein in each case identified by a green node border. See also Tables S1, S2, and S3.

Large overlaps between hypergravity and microgravity genes occurred in the concordant directions (137 commonly upregulated genes and 52 commonly downregulated genes between conditions; Figure 4A). Genes commonly upregulated were involved in processes related to the cell cycle, DNA modification, and ubiquitin-mediated catabolism, whereas commonly downregulated genes were enriched for immune process-related GO terms (Figure 4B). The corresponding protein network of commonly upregulated gravity genes, in particular, was heavily enriched with protein-protein interactions (Figure 4D), with F14H3.6 again identified as the top hub component (Figure 4E). Consistent with TF analysis of hypergravity and microgravity upregulated genes in separation, commonly upregulated gravity genes were also under the predicted control of *efl-1* and *efl-2* (Figure 4C). Thus, these E2F-like TFs appear to represent key regulatory candidates of *C. elegans* transcriptional response to altered gravitational load.

The degree of overlap between genes discordantly regulated by hypergravity and microgravity (i.e., regulated in opposing directions) was less pronounced (Figure 4A). Genes downregulated by hypergravity but upregulated by microgravity were not detectably enriched for GO BP terms or putative protein-protein interactions (Figure 4). However, a distinct biological profile was observed specifically for genes upregulated by hypergravity but downregulated by microgravity. Notably, these genes were enriched for metabolic-related biological processes (Figure 4B), with their corresponding protein network also highly enriched in protein-protein interactions, even when only very strong interactions were considered (Figure 4D). In this case, the top hub component was identified as *hpd-1*, a key enzyme in the degradation of tyrosine (Figure 4E). Moreover, among the predicted TFs of this particular gene set was *dpy-27* (Figure 4C), which is putatively involved in the regulation of growth and body fat metabolism downstream of the TOR complex 2 pathway (UniProt Consortium, 2019).

## Discussion

Exploring and colonizing deep space is a primary aim of the modern space era. In addition to technical challenges, effective countermeasures against the negative health effects of extended spaceflight must be developed. Here we employed *C. elegans* to establish specific transcriptional responses to microgravity versus hypergravity, extending current knowledge of the putative mechanisms underpinning adaptations to altered gravity. We further exploit both predictive network and transcription factor analyses to define candidate molecules that might offer promising mechanistic targets for expediting understanding of, and preventive measures against, microgravity-related health decline.

### Reproducible Gene Signatures of Micro- and Hypergravity Adaptation

Corroborating previous spaceflight studies in worms (Adenle et al., 2009; Higashibata et al., 2016) and rodents (Blaber et al., 2017; Kuznetsov et al., 2019), across spaceflight missions we found consistently upregulated genes associated with altered rates of cell cycle, DNA modification, and actin cytoskeleton/microtubule (a major gravity-sensitive constituent of the cytoskeleton; Papaseit et al., 2000) regulation. Genes enriched for ubiquitin-dependent protein degradation were also consistently upregulated by spaceflight conditions, consistent with observations in space-flown rodent liver (Blaber et al., 2017) and skeletal muscle tissue (Nikawa et al., 2004), and human skeletal muscle using ground-based spaceflight analogs (bedrest/immobilization; Fernandez-Gonzalo et al., 2020; Reich et al., 2010). Although counter to earlier reports of unaltered bulk protein degradation in space-flown *C. elegans* (Etheridge et al., 2011), low-level increases in ubiquitin-proteasome mediated breakdown could be protective against cytotoxic increases in protein aggregates, as occurs during simulated microgravity (Aleshcheva et al., 2013) and animal aging (Melentijevic et al., 2017), a pathophysiological analog of microgravity. Interestingly, our findings extend the microgravity-associated gene profile to include a reproducible downregulation of neuropeptide signaling, indicative of impaired neuronal function. Although poorly studied in higher organisms, recent reports of space-flown mouse liver show reduced neuropeptide gene expression profiles, which was not observed in kidney tissue (Hammond et al., 2018), perhaps indicative of tissue-specific neuropeptide signaling dysregulation during spaceflight. Moreover, emerging evidence in astronauts indicates that brain white matter changes occur during space travel (Lee et al., 2019), perhaps suggesting abnormal neuronal transcriptional signatures and associated physiological changes might also be relevant in people. Regardless, the observed molecular profile herein directly adheres with the negative effects of space travel on neuromuscular and central nervous system functions (Fitts et al., 2010; Newberg and Alavi, 1998). We also note that, although reproducible microgravity gene profiles were only found when small expression changes were considered, subtle yet significant fold-changes is likely a true feature of spaceflight adaptation (Higashibata et al., 2016; Selch et al., 2008). Indeed, this reflects the modest but clinically important health effects of spaceflight and is comparable with the magnitude of physiological and gene changes observed with unloading-related health defects in humans on Earth (Adams et al., 2003).

Analysis of the hypergravity transcriptome profile revealed, similarly to microgravity, upregulation of genes enriched for cell cycle processes. Upregulation of cell cycle genes has also been reported in mouse hippocampal tissue following exposure to rotation plus hypergravity (Del Signore et al., 2004). *In vivo* sensitivity to progressive hypergravity is also shown, with additional nuclear and metabolic program increases only at the highest 15 × *g* forces. A similar progressive gene response was observed for downregulated pathways: suppressed innate immunity featured across hypergravity and, although not attributable to specific biological functions, 15 × *g* alone reduced genes involved in “translation processes”, all hub components of which were found to represent ribosomal complex proteins. This observation corroborates earlier reports of (mito)ribosomal gene downregulation as a molecular feature of chronic hypergravity exposure in fruit flies (Hateley et al., 2016). Thus, these data support a role for hypergravity in suppressing immune system responsiveness, a characteristic also common to microgravity exposure (Crucian et al., 2018), as well as ribosomal complex functioning.

### A Neuronal Metabolic Stress Response as a General Micro- and Hypergravity Adaptation

A central component of our analysis was to compare and contrast the micro- and hypergravity transcriptional response, on the premise that the most robust gene signatures of gravity adaptation might be the inverse of one another and/or reproducible across conditions. Interestingly, we found that the majority of overlapping gene changes with micro- and hypergravity actually occurred in the same direction. The fact that concordance is dominant seems to suggest that any change from 1 × *g* gravity takes *C. elegans* out of their native environment and disrupts their homeostasis, triggering a similar systemic response independently of the direction in which gravity is altered. The majority of concordant differentially expressed upregulated genes for micro-/hypergravity are involved in cell cycle, actin cytoskeleton regulation, DNA modification, and ubiquitin processes, whereas genes involved in immunity pathways are commonly downregulated. Interestingly, this corroborates findings in rat mammary tissue where regulation of cell cycle, actin cytoskeleton, and DNA modification (specifically chromatin modification) genes was a common feature of both hypergravity and spaceflight exposure (Casey et al., 2015). Our predictive transcription factor and network analyses further indicate common transcriptional and hub regulators across these broad gravity gene profiles. Although the biological implications of the full list of top-ranked hub components and predicted transcription factors is beyond the scope of this discussion, these should serve as a useful tool for future hypothesis-driven work.

The most highly connected hub component within the protein interaction network for genes upregulated across gravities was F14H3.6. The biological function of F14H3.6 is poorly characterized, but this gene is expressed in neuronal sheath cells under transcriptional control by *daf-16*/FOXO (Rizki et al., 2011), which has long been associated with the spaceflight response (Honda et al., 2012, 2014; Selch et al., 2008). Transcription factors predicted to regulate these upregulated genes also have neuronal roles: those identified are E2F transcription factors with expression in ventral (*efl-1*) and ventral/dorsal (*efl-2*) nerves (Harris et al., 2020). On Earth, *efl-1* mutants also display ectopic neuronal *unc-4* expression, another TF involved in nervous system development and synapse structure/activity (Zheng et al., 2013) and, interestingly, *efl-1* also interacts with *daf-16*/FOXO to coordinate cellular senescence (Xie et al., 2014). Additionally, *egl-21* and *nsy-7* returned as the top hub component and predicted transcription factor, respectively, for microgravity responses specifically. Consistent with a neuronal phenotype, *egl-21* mutants display impaired production of several neuropeptides (Jacob and Kaplan, 2003) and, again, *egl-21* is repressed in neuronal-specific *daf-16* mutants (Nagashima et al., 2019), further implicating a close gravity-neuronal-FOXO functional link.

Lastly, overlapping gene profiles with inverse expression changes between microgravity and hypergravity might, teleologically, represent strong mechanistic candidates for gravity adaptation. We found corresponding genes with differential downregulation in microgravity but upregulation in hypergravity were enriched for metabolism-related GO terms, indicative of inverted metabolic responses between gravity stimuli. Inverse metabolic gene expression changes following hypergravity versus spaceflight exposure also appears as a molecular characteristic of rat mammary tissue (Casey et al., 2015). Network-driven analysis revealed *hpd-1*, a tyrosine degrading enzyme, as the most highly connected hub component. Mutant *hpd-1* animals exhibit increased cellular protein aggregates, leading to metabolic disease (Ferguson et al., 2010) and, as with other gravity-related hub components, *hpd-1* is a transcriptional target of *daf-16*/FOXO (Lee et al., 2003; Murphy, 2006).

The transcriptomic evidence presented herein thus strongly indicates further putative features of the gravity response, namely, alterations in neuronal structure and signaling, that could account for several of the well-known phenotypes associated with altered gravitational loading in higher organisms (Demontis et al., 2017; Frett et al., 2016; Genchi et al., 2016). Since we cannot distinguish tissue-specific responses, it remains possible that neuronal and metabolic gene changes are entirely independent, whereby altered metabolism is an organism-wide, non-tissue specific adaptation to varying gravity. However, because neurons are one of the body's most highly metabolic tissues (Camandola and Mattson, 2017), any organism-wide metabolic perturbation might first be expected to present in neuronal tissue. Importantly, all identified network hubs and transcription factors have established regulatory functions within the *daf-2*/insulin > *daf-16*/FOXO signaling cascade. It is, therefore, plausible that the *in vivo* response to both increased and decreased gravitational load is underpinned by changes in neuronal function that likely respond to *daf-16*/FOXO-sensitive pathways to effect alterations in neuron metabolism as part of a general, gravity-dependent stress response. Indeed, the *daf-16*/FOXO pathway has been implicated in physiological responses to spaceflight (Honda et al., 2012; Szewczyk et al., 2008) and hypergravity (Kim et al., 2007).

### Conclusion

Here, we contrasted the microgravity and hypergravity transcriptomes of *C. elegans* to provide further insights into the molecular adaptations to altered gravitational load. Micro- and hypergravity responsive gene signatures are consistently characterized by network hubs and predicted transcription factors with terrestrial roles in neuronal function and/or cellular metabolism which, in turn, are consistently linked to *daf-16*/FOXO regulation. Given that *daf-16* functions, in part, as a stress response element that controls cellular metabolism to influence health and longevity (Gurkar et al., 2018), we propose that *daf-16*-induced metabolic reprogramming of neurons might represent a central facet of altered gravity. In this context, and because our findings corroborate available data in rodents, the list of molecular features presented herein should serve as a strong platform for future hypothesis-driven work to understand the mechanisms of microgravity-related maladaptation, accelerating development of targeted therapeutics against health decline in space for forward-translation into mammals and, ultimately, humans.

### Limitations of the Study

The microarray datasets utilized in this study each contain gene expression data that was generated using total RNA extracted from whole worms. Thus, despite *C. elegans* being the simplest *in vivo* model organism, our data cannot distinguish between tissue-specific transcriptional responses to altered gravity. Although tissue-specific transcriptomics are possible in *C. elegans* (Kaletsky et al., 2018), no accessible cell- or tissue-specific transcriptomic datasets from *C. elegans* were available in the contexts of hypergravity or spaceflight. Future studies could address this to facilitate cross-species interpretations and provide greater resolution of organ-(in)dependent transcriptional changes, an important consideration given the wide-ranging effects of altered gravity across various physiological systems (Demontis et al., 2017; Frett et al., 2016; Genchi et al., 2016). Additionally, our findings establish multiple hypotheses, and several associated gene signatures have been independently validated via RT-/qPCR in microgravity samples derived from the same missions (Higashibata et al., 2006, 2016; Honda et al., 2012), a subset of which has also been shown to display corresponding proteomic changes (Higashibata et al., 2006, 2016). Nonetheless, future targeted quantitative assessment of the molecular changes reported herein are required to confirm the presently reported expression changes during altered gravity.

### Resource Availability

#### Lead Contact

Further information and requests for resources and reagents should be directed to and will be fulfilled by the Lead Contact, Timothy Etheridge (t.etheridge@exeter.ac.uk).

#### Materials Availability

This study did not generate new unique reagents.

#### Data and Code Availability

This study did not generate any new datasets but analyzed datasets contained within the NASA GeneLab public data repository (genelab.nasa.gov). Accession numbers for all datasets analyzed are given in Table 1.

## Methods

All methods can be found in the accompanying Transparent Methods supplemental file.

## Supplemental Information

Document S1. Transparent Methods

Table S1. Complete Lists of Differentially Expressed Genes for Each Comparison, Related to Figures 1–4

Table S2. Complete Lists of Enriched Gene Ontology Biological Process Terms for Each List of Differentially Expressed Genes, Related to Figures 1–4

Table S3. Hub Statistics Obtained from Protein-Protein Network Analysis of Each Differentially Expressed Gene List, Related to Figures 1–4
