# Supplementary material for: Comparative Transcriptomics Identifies Neuronal and Metabolic Adaptations to Hypergravity and Microgravity in Caenorhabditis elegans
Source: iScience. 2020 Nov 25;23(12):101734. doi: 10.1016/j.isci.2020.101734 (PMC7756135; doi:10.1016/j.isci.2020.101734)
Supplement: Document S1. Transparent Methods [file mmc1.pdf]

iScience, Volume *23*

Supplemental Information

Comparative Transcriptomics Identifies Neuronal and Metabolic Adaptations to Hypergravity and Microgravity in *Caenorhabditis elegans*

Craig R.G. Willis, Nathaniel J. Szewczyk, Sylvain V. Costes, Ingrid A. Udranszky, Sigrid S. Reinsch, Timothy Etheridge, and Catharine A. Conley

Transparent Methods

Data description

All datasets used in the current work were acquired from the NASA GeneLab public data repository (genelab.nasa.gov). The gene expression data for hypergravity (GLDS-190, Szewczyk et al., n.d.) were previously published as part of a “meta-analysis” (Kim et al., 2001). To perform the hypergravity studies, separate but identically prepared subcultures of mixed stage *C. elegans* strain CC1 were grown in 75 cm^2 cell culture flasks each containing 25 mL of *C. elegans* Maintenance Medium (CeMM), as described (Szewczyk et al., 2003). For each control/hypergravity condition, twelve subcultures per condition each with an approximate density of 10,000 worms per mL of media were concurrently subject to four days of either stationary culture (control) or 5xg, 10xg, or 15xg hypergravitational force on the 8.84 meter, 20-G centrifuge at NASA Ames Research Center. The different g levels were achieved by placing experimental cultures at an appropriate position on the centrifuge as calculated to achieve the desired amount of hypergravitational force in each instance, with 1xg stationary cultures placed in the same room to control for vibration plus other environmental factors, and procedures conducted at room temperature throughout. Total RNA was subsequently extracted from whole worms using Tri Reagent LS, with 1 mL of culture used to produce RNA for each prep. The cDNA of control and experimental worms was then prepared and labelled with Cy5 (red) and Cy3 (green) fluorescent dye, respectively, via reverse transcription and analysed on non-commercial two-channel microarrays (Stanford, US) as described (Kim et al., 2001; Reinke et al., 2000), with three arrays generated per level of the hypergravity treatment. The experimental procedures associated with each spaceflight dataset used here (GLDS-113, Higashibata and Hashizume, n.d.a; GLDS-112, Higashibata and Hashizume, n.d.b; GLDS-41, Higashibata and Hashizume, n.d.c) can be found in previous work (Higashitani et al., 2009; Szewczyk et al., 2008) and are summarised in Table 1. In all three cases, total RNA was isolated from whole worms using Isogen Reagent, with Cy3 labelled cRNA then prepared and analysed on the *C. elegans* Oligo Microarray 44 k version 2.0 platform (Agilent Technologies, Santa Clara, CA, USA) as described (Higashibata et al., 2016). Within each generated spaceflight dataset, arrays corresponding to either space-flown worms or 1xg ground control worms were identified and extracted for subsequent pre-processing/ analyses.

Pre-processing of microarray data

Arrays were processed on a study-by-study basis using the Linear Models for Microarray Analysis (LIMMA) package in R (Ritchie et al., 2015). For GLDS-190, flagged spots on each array were first removed, after which mean foreground intensities were corrected for median

background intensities using the normexp method with an offset of 50. Within-array normalisation was then performed using the global loess method, followed by between-array normalisation using the quantile method. Control probes and probes without a unit of expression for all samples were subsequently removed, with the expression of probes corresponding to the same WormBase Gene ID then averaged. For each spaceflight dataset, mean foreground intensities were similarly corrected for median background intensities using the normexp method and with an offset of 50. Between-array normalisation was then applied using the quantile method. Control and consistently non-expressed probes (above background) were subsequently removed, after which expression of probes corresponding to the same WormBase Gene ID was averaged. Expression values were also averaged across technical replicates where appropriate (dataset GLDS-41). Finally, we filtered each of the four datasets for those WormBase Gene ID's present across every one of them, to obtain a unified set of 9761 genes for appropriate downstream analyses.

Identifying differential expression

For each study, differential expression was inferred between gravity condition(s) and corresponding 1xg controls by fitting a linear model to each gene using an empirical Bayes approach, as implemented in the R LIMMA package (Ritchie et al., 2015; Smyth, 2004). In each case, array weights were included into linear models to account for relative array quality in accordance with the design of each study (Ritchie et al., 2006). Differentially expressed genes were subsequently defined as those with a Benjamini-Hochberg (BH) corrected $P \leq 0.1$.

Establishing overlaps across gravity-regulated genes

To determine common and uniquely regulated genes across different hypergravity levels we utilised the rank-rank hypergeometric overlap algorithm (Cahill et al., 2018; Plaisier et al., 2010), in which genes were ranked on sign of differential expression multiplied by the negative log10 of their BH-corrected *P*-value. Commonly regulated hypergravity genes were subsequently defined as those significantly differentially expressed in both conditions *and* present within the optimal statistically significant overlapping gene set between conditions. Uniquely regulated hypergravity genes were then defined as those significantly differentially expressed in a single condition *and not* present within the optimal statistically significant overlapping gene set between conditions. Significance of overlap between genes differentially regulated across microgravity datasets was determined using a scalable Fisher Exact algorithm for testing multi-set interactions, as implemented in the R SuperExactTest package (Wang et al., 2015). Significance of overlap between genes differentially regulated in hypergravity *vs.* microgravity was elucidated using the standard Fisher Exact Test. Overlaps were considered statistically significant when $P < 0.05$.

Functional annotation of gravity gene changes

Functional characteristics of gravity-regulated gene sets were derived by testing their enrichment for Gene Ontology Biological Process terms, using the clusterProfiler package in R (Yu et al., 2012). The background gene list in each instance comprised all genes that were found present in each dataset after pre-processing (i.e. the 9761 genes tested for differential expression). Terms with a BH-corrected *P*-value < 0.05 were subsequently defined as being enriched. For visualisation purposes, enriched Gene Ontology terms were summarised by shorter 'representative' term lists by removing redundant terms using the online REVIGO tool (Supek et al., 2011).

Protein network analysis of gravity gene changes

Protein networks were constructed for gravity-regulated gene sets using the online Search Tool for the Retrieval of Interacting Genes/Proteins (STRING; Szklarczyk et al., 2019). Notably, STRING quantifies the strength of any given protein-protein interaction by a confidence score, of which provides the approximate probability that a link exists between a given protein pair. We thus initially constructed weighted protein networks in which all potential protein-protein interactions were included, regardless of their strength (i.e. confidence score > 0). We then considered network enrichment for protein-protein interactions across a range of different strength cut-offs, namely: (i) weak and above (confidence score > 0.15); (ii) strong and above (confidence score > 0.4), and; (iii) very strong and above (confidence score > 0.7). In any instance, networks were considered significantly enriched in protein-protein interactions when $P < 0.05$. Finally, we deduced hub components in protein networks using the Kleinberg hub score metric (Csardi and Nepusz, 2006; Kleinberg, 1999), calculated as the principle eigenvector of AA^T, where A is the network adjacency matrix. Network components with a Kleinberg hub score > 0.8 were subsequently defined as hubs. Where applicable, network visualisations were generated using Cytoscape (Shannon et al., 2003).

Uncovering putative transcriptional regulators of gravity gene changes

Putative TFs were identified by testing gravity-regulated gene sets for enriched upstream regulators using the WormEnrichr web service (Kuleshov et al., 2019). In particular, we queried the TF2DNA database (2018 version), which contains organism-specific TF target gene sets based on TF binding motifs (Kuleshov et al., 2019; Pujato et al., 2014). Enrichment significance was assessed using the Enrichr combined score: a robust metric for quantifying enrichment that is obtained by multiplying the log of the Fisher exact enrichment *P*-value by the Z-score of the deviation from the expected enrichment rank. TFs with a confidence score > 4 were subsequently considered as enriched.

Supplemental References

Cahill, K.M., Huo, Z., Tseng, G.C., Logan, R.W., and Seney, M.L. (2018). Improved identification of concordant and discordant gene expression signatures using an updated rank-rank hypergeometric overlap approach. Sci. Rep. *8*, 9588.

Csardi, G., and Nepusz, T. (2006). The igraph software package for complex network research. InterJournal *Complex Sy*, 1695.

Higashibata, A., and Hashizume, T. (n.d.a). Expression Data from International C. elegans Experiment 1st (ICE-FIRST).

Higashibata, A., and Hashizume, T. (n.d.b). Microgravity effect on C. elegans N2/VC (CERISE, 4 days).

Higashibata, A., and Hashizume, T. (n.d.c). Microgravity effect on C. elegans N2/VC (CERISE, 8days).

Higashibata, A., Hashizume, T., Nemoto, K., Higashitani, N., Etheridge, T., Mori, C., Harada, S., Sugimoto, T., Szewczyk, N.J., Baba, S.A., et al. (2016). Microgravity elicits reproducible alterations in cytoskeletal and metabolic gene and protein expression in space-flown Caenorhabditis elegans. Npj Microgravity *2*, 15022.

Higashitani, A., Hashizume, T., Sugimoto, T., Mori, C., Nemoto, K., Etheridge, T., Higashitani, N., Takanami, T., Suzuki, H., Fukui, K., et al. (2009). C. elegans RNAi space experiment (CERISE) in Japanese Experiment Module KIBO. Biol. Sci. Space. *23*, 183–187.

Kim, S.K., Lund, J., Kiraly, M., Duke, K., Jiang, M., Stuart, J.M., Eizinger, A., Wylie, B.N., and Davidson, G.S. (2001). A Gene Expression Map for Caenorhabditis elegans. Science *293*, 2087–2092.

Kleinberg, J.M. (1999). Authoritative sources in a hyperlinked environment. J. ACM *46*, 604–632.

Kuleshov, M. V, Diaz, J.E.L., Flamholz, Z.N., Keenan, A.B., Lachmann, A., Wojciechowicz, M.L., Cagan, R.L., and Ma'ayan, A. (2019). modEnrichr: a suite of gene set enrichment analysis tools for model organisms. Nucleic Acids Res. *47*, W183–W190.

Plaisier, S.B., Taschereau, R., Wong, J.A., and Graeber, T.G. (2010). Rank–rank hypergeometric overlap: identification of statistically significant overlap between gene-expression signatures. Nucleic Acids Res. *38*, e169.

Pujato, M., Kieken, F., Skiles, A.A., Tapinos, N., and Fiser, A. (2014). Prediction of DNA binding motifs from 3D models of transcription factors; identifying TLX3 regulated genes. Nucleic Acids Res. *42*, 13500–13512.

Reinke, V., Smith, H.E., Nance, J., Wang, J., Van Doren, C., Begley, R., Jones, S.J., Davis, E.B., Scherer, S., Ward, S., et al. (2000). A global profile of germline gene expression in C.

elegans. Mol. Cell *6*, 605–616.

Ritchie, M.E., Diyagama, D., Neilson, J., van Laar, R., Dobrovic, A., Holloway, A., and Smyth, G.K. (2006). Empirical array quality weights in the analysis of microarray data. BMC Bioinformatics *7*, 261.

Ritchie, M.E., Phipson, B., Wu, D., Hu, Y., Law, C.W., Shi, W., and Smyth, G.K. (2015). limma powers differential expression analyses for RNA-sequencing and microarray studies. Nucleic Acids Res. *43*, e47.

Shannon, P., Markiel, A., Ozier, O., Baliga, N.S., Wang, J.T., Ramage, D., Amin, N., Schwikowski, B., and Ideker, T. (2003). Cytoscape: a software environment for integrated models of biomolecular interaction networks. Genome Res. *13*, 2498–2504.

Smyth, G.K. (2004). Linear Models and Empirical Bayes Methods for Assessing Differential Expression in Microarray Experiments. Stat. Appl. Genet. Mol. Biol. *3*.

Supek, F., Bošnjak, M., Škunca, N., and Šmuc, T. (2011). REVIGO Summarizes and Visualizes Long Lists of Gene Ontology Terms. PLoS One *6*, e21800.

Szewczyk, N.J., Conley, C.A., and Wang, J. (n.d.). Worms spun in centrifuge at elevated g values.

Szewczyk, N.J., Kozak, E., and Conley, C.A. (2003). Chemically defined medium and Caenorhabditis elegans. BMC Biotechnol. *3*, 19.

Szewczyk, N.J., Tillman, J., Conley, C.A., Granger, L., Segalat, L., Higashitani, A., Honda, S., Honda, Y., Kagawa, H., Adachi, R., et al. (2008). Description of International Caenorhabditis elegans Experiment first flight (ICE-FIRST). Adv. Space Res. *42*, 1072–1079.

Szklarczyk, D., Gable, A.L., Lyon, D., Junge, A., Wyder, S., Huerta-Cepas, J., Simonovic, M., Doncheva, N.T., Morris, J.H., Bork, P., et al. (2019). STRING v11: protein–protein association networks with increased coverage, supporting functional discovery in genome-wide experimental datasets. Nucleic Acids Res. *47*, D607–D613.

Wang, M., Zhao, Y., and Zhang, B. (2015). Efficient Test and Visualization of Multi-Set Intersections. Sci. Rep. *5*, 16923.

Yu, G., Wang, L.-G., Han, Y., and He, Q.-Y. (2012). clusterProfiler: an R package for comparing biological themes among gene clusters. OMICS *16*, 284–287.
